# Case Report: Lithoplasty-Assisted Trans-Axillary Transcatheter Aortic Valve-in-Valve Implantation

**DOI:** 10.3389/fcvm.2021.747588

**Published:** 2021-10-20

**Authors:** Alfredo Giuseppe Cerillo, Matteo Pennesi, Luisa Iannone, Giorgia Giustini, Paolo de Cillis, Renato Valenti, Niccolò Marchionni, Pierluigi Stefano

**Affiliations:** ^1^Unit of Cardiac Surgery, Careggi University Hospital, Florence, Italy; ^2^Interventional Cardiology, Careggi University Hospital, Florence, Italy; ^3^General Cardiology, Careggi University Hospital, Florence, Italy; ^4^Department of Clinical and Experimental Medicine, University of Florence School of Medicine, Florence, Italy

**Keywords:** transcatheter aortic valve implantation (TAVI), shockwave, trans-subclavian approach, multi-district atherosclerosis, redo cardiac surgery

## Abstract

We present the case of a severely symptomatic patient with a malfunctioning aortic bioprosthesis and severe multidistrict atherosclerosis that was addressed to our unit for transcatheter valve-in-valve implantation. The imaging and clinical assessment that led to the selection of the access route is discussed.

## History of Presentation

An 84-year-old man was addressed to our outpatient clinic for treatment of a degenerated aortic bioprosthesis. The patient had severe aortic stenosis and quickly ingravescent dyspnea (NYHA class III). He had multiple major comorbidities and the STS score was 12.043%, but he was still active and was heavily symptomatic. After multidisciplinary discussion, the Heart Team recommended screening for transcatheter aortic valve-in-valve implantation.

## Medical History

The patient had type 2 diabetes and hypertension. He had had a transient ischemic attack in 1998 and a stroke with mild residual hemiparesis in 2001. After the stroke, he underwent several investigations that led to the diagnosis of paroxysmal atrial fibrillation, severe aortic stenosis, and single vessel disease of the right coronary artery. In 2002, he underwent aortic valve replacement with a 23-mm Carpentier Edwards Magna bioprosthesis plus saphenous vein graft for the posterior descending artery.

**Figure 1:**
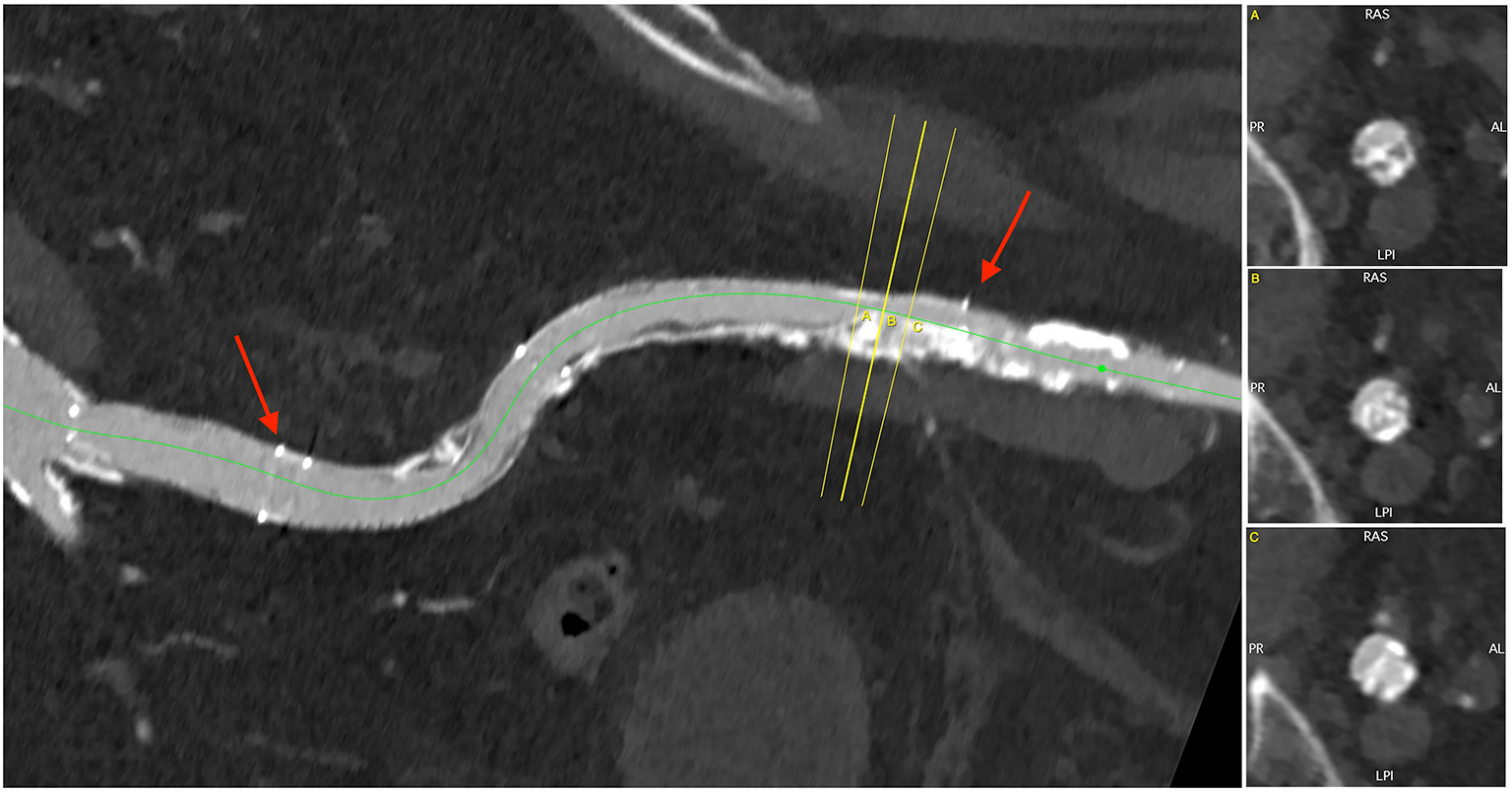
The right iliac artery was chronically occluded, and an iliofemoral bypass (red arrows) was nearly occluded at the distal anastomosis.

**Figure 2:**
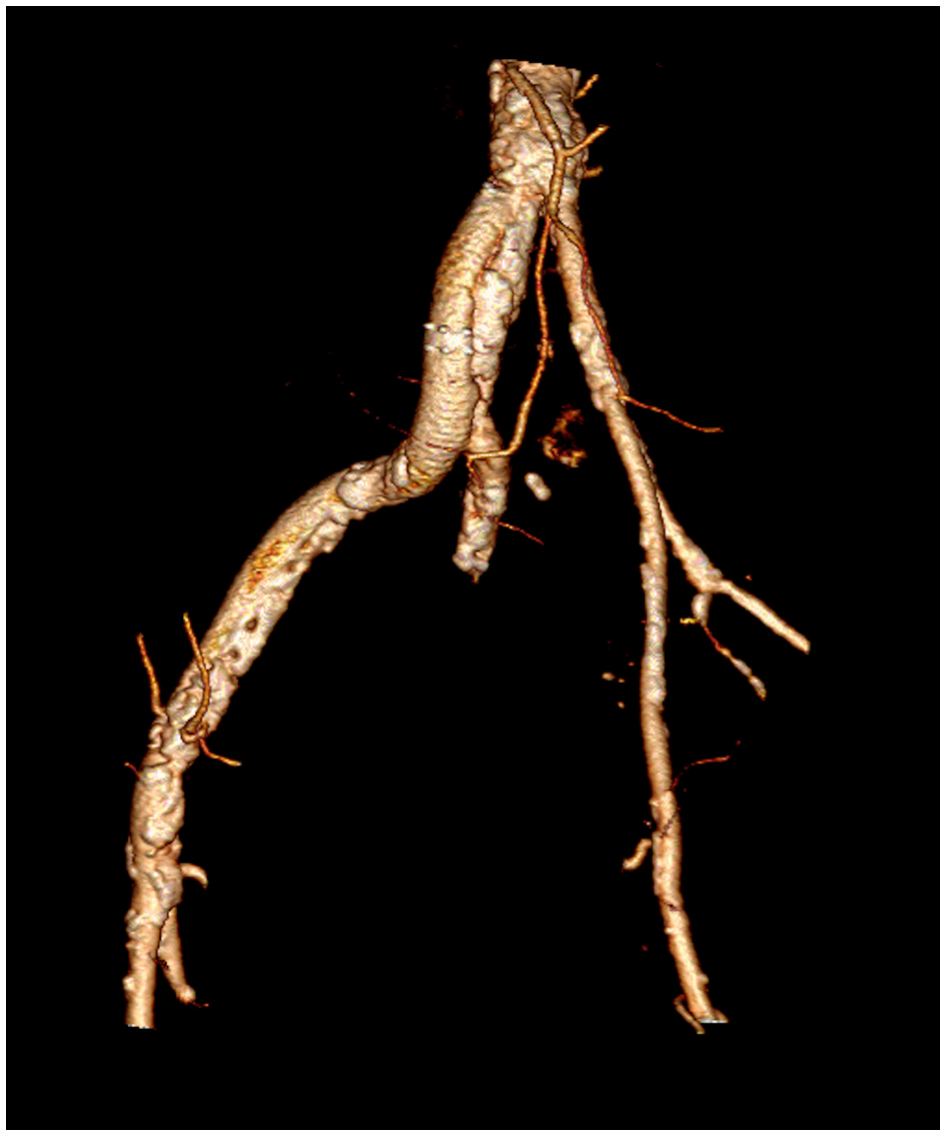
3D reconstruction of the iliofemoral axes, demonstrating the hypoplasia of the left axis and massive calcific involvement of the right axis.

**Figure 3:**
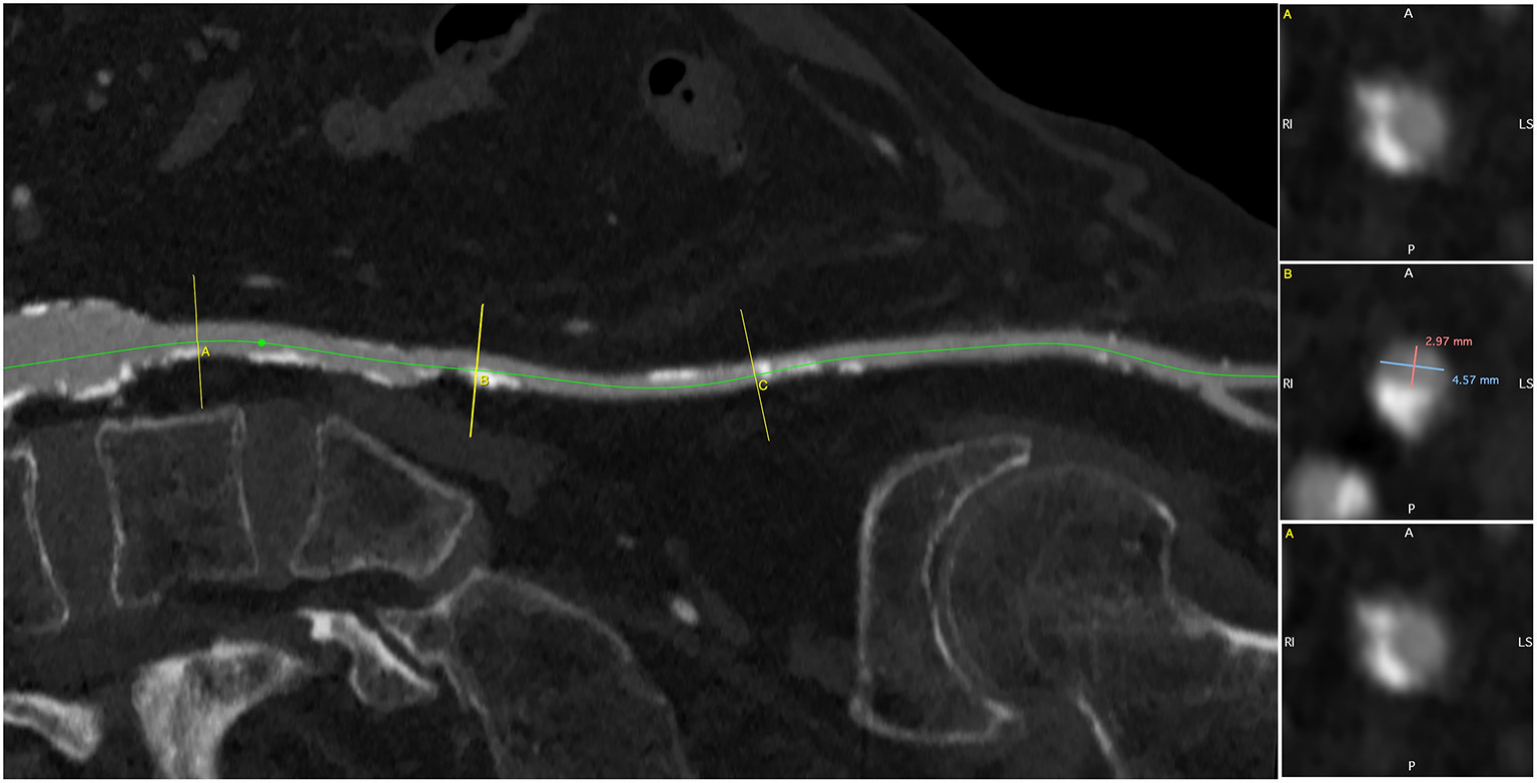
The left iliofemoral axis was diffusely hypoplastic (<5 mm), with multiple calcific stenoses.

**Figure 4:**
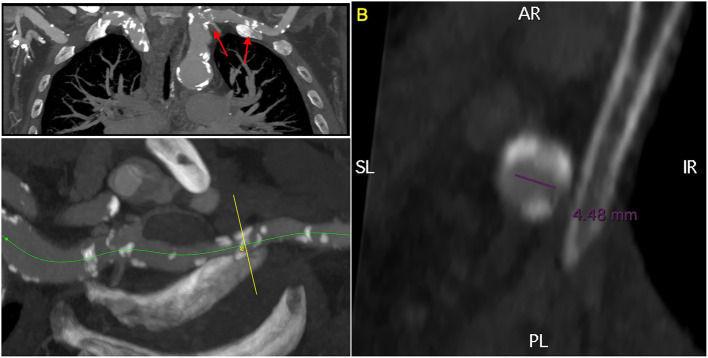
3D multiplanar reconstruction of the left subclavian artery, demonstrating moderate calcifications and a focal stenosis with a residual lumen of <5 mm. The red arrows indicate the target lesions of the lithoplasty.

**Figure 5:**
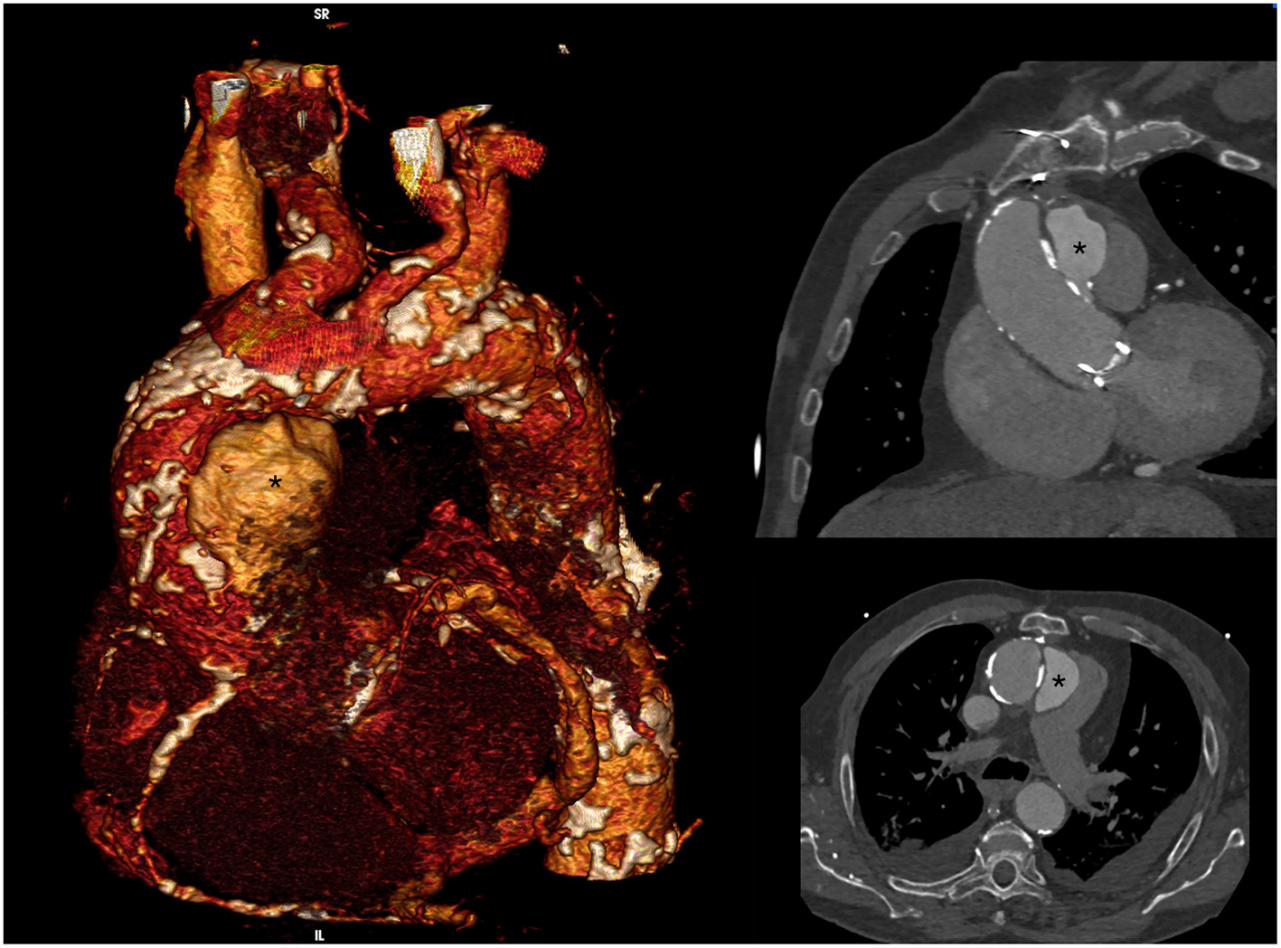
Volume rendering and 3D MPR images of the ascending aorta and aortic arch, demonstrating the presence of severe calcific atherosclerosis and a big aortic pseudoaneurysm (*).

The patient had multiple comorbidities:

- Major functional limitation due to poor mobility. The patient had had poliomyelitis with residual hypoplasia of the left lower limb. In 2019, he underwent orthopedic surgery for a fracture of the right leg, and since then he had to move on a wheelchair.
- Severe multidistrict atherosclerosis, including severe stenosis of the right and left iliofemoral axes. A previous right iliofemoral bypass was occluded at the distal anastomosis. The left iliac and femoral artery were hypoplastic and stenotic (Figures 1–**4**).
- Moderate left ventricular dysfunction (LVEF 45%) and pulmonary hypertension.
- Chronic renal failure.
- Chronic lung disease: the patient was on domiciliar oxygen therapy since 2014, but there was no recent pneumologic evaluation.
- Severe calcific atherosclerosis of the ascending aorta and aortic arch with a pseudoaneurysm of the ascending aorta at the level of the previous aortotomy (**Figure 5**).

Despite the functional limitation and the important comorbidities, the patient was active and had a rich relational life. He was heavily symptomatic and had experienced a sudden worsening of the dyspnea. After in-depth multidisciplinary discussion, the heart team decided for transcatheter valve-in-valve implantation.

## Investigations

The echocardiography confirmed the presence of prosthetic structural valve degeneration with severe aortic stenosis. The aortic valve area was 0.7 cm^2^, and the mean transprosthetic gradient was 53 mmHg. At CT, the prosthesis was severely calcified. There was pulmonary hypertension (PAP 65 mmHg).

The patient underwent pneumologic evaluation including high-resolution chest CT and right heart catheterization. The conclusion was that he had a moderate chronic obstructive pulmonary disease, and that his dyspnea was mainly cardiac in origin.

At CT, the right and left iliofemoral access were considered not feasible (Figures 1–3). The left subclavian artery showed a moderate atherosclerotic involvement with a focal stenosis at the thoracic outlet (residual lumen <5 mm, Figure 4). The right subclavian and trans-carotid access were discarded for the presence of major tortuosity and calcification of the innominate artery, left carotid artery, and aortic arch. The transapical and transaortic accesses were feasible, but they were considered a poor option for this delicate patient that would have hardly tolerated the general anesthesia and the surgical trauma related to the thoracotomy. Also, chest re-entry was at risk for the presence of an aortic pseudoaneurysm (Figure 5, Supplementary Video 1).

## Management

The patient underwent lithoplasty-assisted trans-axillary aortic valve-in-valve implantation with a 26-mm Medtronic Evolut prosthesis. Under loco-regional anesthesia, the left axillary artery was isolated at the deltoid–pectoralis groove and exposed with a soft-tissue retractor. A 4/0 Prolene purse string was prepared and an 8F vascular sheet was inserted, through which a 6.5 × 60 mm lithoplasty balloon (Shockwave Medical, Freemont, CA, USA) was advanced across the lesion and inflated to 4 atm. Pulse pressure waves were delivered for 30″ × 4 cycles (Supplementary Video 2). Two additional cycles were delivered at the main curvature of the left subclavian artery to facilitate the navigation of the transcatheter valve (Supplementary Video 3). The malfunctioning bioprosthesis was crossed and a 26-mm Evolut valve was successfully advanced and deployed on a pre-shaped stiff wire. The delivery system was removed and the purse-string was tied. The control angiography showed a patent left subclavian artery with no dissection or extravasation. The aortic pseudo-aneurysm was not affected by the procedure (Supplementary Videos 4, 5). A pace-maker was implanted for a prolonged pause at ECG monitoring. The patient returned to the general ward and was discharged to a rehabilitation facility on post-operative day 7 after an uncomplicated recovery. At discharge, the control echocardiogram showed a mean transprosthetic gradient of 8 mmHg and mild residual aortic regurgitation.

## Discussion

Vascular lithoplasty—disruption of calcium within vascular plaques with circumferential pulse pressure waves emitted by a balloon catheter—has been successfully used to treat different conditions, including peripheral artery disease (1) and coronary artery disease (2). Recently, vascular lithoplasty has been used to facilitate vascular access during transfemoral transcatheter aortic valve implantation (TAVI) (3) or endovascular aneurysm repair (EVAR) (4). To our knowledge, lithoplasty-assisted trans-subclavian TAVI has not been previously reported.

TAVI can be performed through several routes—trans-femoral, trans-subclavian, trans-carotid, trans-aortic, trans-apical, and trans-caval. However, the surgical approaches requiring general anesthesia and chest opening are more invasive, and there is evidence that they expose the patient to higher 30-day and 1-year mortality rates (5). Our patient was not a candidate for open heart surgery. Moreover, he had several relative contraindications to general anesthesia and intubation, including moderate COPD, moderate left ventricular dysfunction, and poor mobility that precluded the trans-aortic and trans-apical approaches. The right and left iliofemoral axes were severely diseased: the left iliac and femoral artery were hypoplastic (<5 mm) as a consequence of previous poliomyelitis, with a diffuse calcific atherosclerotic involvement. The right external iliac artery was occluded, and a previous iliofemoral bypass was also nearly occluded at the distal anastomosis, where a bulky calcification of the native vessel was present.

As the transfemoral approach, the trans-axillary approach can be performed under loco-regional anesthesia (ultrasound guided brachial plexus block). In our patient, the left subclavian artery was diffusely atherosclerotic and calcific, but had a single focal stenosis <2 cm long and had overall good dimensions. We therefore decided to attempt a lithoplasty-assisted trans-subclavian approach under loco-regional anesthesia. Although the advancement of the device required some maneuver, the stenosis was crossed and the procedure was successful. Pulse pressure waves were delivered also at the curvature of the subclavian artery to soften the calcific plaques. This allowed to straighten the vessel and facilitated the navigation of the transcatheter valve.

## Follow-Up

At the 6-month follow-up, the patient was alive and well. The symptomatic status had improved to NYHA class II and the echocardiography showed a normo-functioning trans-catheter valve.

## Conclusions

The lithoplasty-assisted trans-axillary access is a feasible option for patients with prohibitive trans-femoral accesses and major contraindications to general anesthesia and/or thoracotomy. The application of pulse pressure waves to the calcific plaques allows to cross focal calcific stenoses and facilitates the navigation through tortuous calcific vessels.

## Learning Objectives

- To recognize the different clinical, pathologic, and anatomical factors that determine the choice of the optimal access route in patients undergoing TAVI.
- To realize the potential usefulness of catheter-based lithoplasty in patients with prohibitive accesses, also in district other than the iliofemoral.

## Data Availability Statement

The original contributions presented in the study are included in the article/Supplementary Material, further inquiries can be directed to the corresponding author.

## Ethics Statement

Ethical review and approval was not required for the study on human participants in accordance with the local legislation and institutional requirements. The patients/participants provided their written informed consent to participate in this study.

## Author Contributions

AC, NM, and PS: study design and conceptualization. AC, LI, and GG: clinical management and planning. AC, MP, PC, and RV: performance of the procedure. AC: writing the paper. MP, LI, GG, PC, RV, NM, and PS: critical revision and acceptance of the paper. All authors contributed to the article and approved the submitted version.

## Funding

This work has been supported by a donation offered to Fondazione A.R. Card Onlus in memory of Marco Sarti by his beloved wife, son, and relatives and friends.

## Conflict of Interest

The authors declare that the research was conducted in the absence of any commercial or financial relationships that could be construed as a potential conflict of interest.

## Publisher's Note

All claims expressed in this article are solely those of the authors and do not necessarily represent those of their affiliated organizations, or those of the publisher, the editors and the reviewers. Any product that may be evaluated in this article, or claim that may be made by its manufacturer, is not guaranteed or endorsed by the publisher.
